# Efficient SqueezeViT: A lightweight vision transformer framework for chest X-ray image classification

**DOI:** 10.1038/s41598-026-47918-4

**Published:** 2026-04-09

**Authors:** Abhinav Maurya, Ashish Lohia, Chirag, Jyoti Yadav, Bharti Panjwani, Vijay Mohan

**Affiliations:** 1 Department of Instrumentation and Control Engineering, Netaji Subhas University of Technology, Sector-3, Dwarka, New Delhi India; 2 Department of Computer Science & Engineering, Shri Madhwa Vadiraja Institute of Technology and Management, Bantakal, Karnataka India; 3 Manipal Institute of Technology, Manipal Academy of Higher Education, Manipal, 576104 Karnataka India

**Keywords:** SqueezeViT, MobileViT, CNN, Chest X ray, Computational biology and bioinformatics, Engineering, Mathematics and computing

## Abstract

This work introduces SqueezeViT (Squeeze Vision Transformers), a compact yet effective architecture based on Vision Transformers (ViT) designed for chest X-ray (CXR) image classification. In contrast to traditional ViT architectures, which are computationally demanding, SqueezeViT employs a novel squeezing procedure that effectively lowers token dimensions without compromising important visual components, leading to expedited inference and decreased memory consumption. The designed model is tested for two commonly used public datasets, NIH Chest X-ray and CheXpert, providing a diverse range of thoracic pathologies. SqueezeViT reduces the number of parameters by 43.2% compared to the baseline MobileViT^1^, and up to 95.4% compared to other state-of-the-art (SOTA) models. The suggested model offers up to 16.5% improvement in the area under the receiver operating characteristic curve (AUROC) compared to SOTA models, and it is, in general, superior to the baseline and effective convolutional neural networks CNNs^2^ in numerous tasks. Such developments make the proposed SqueezeViT approach an attractive option for a wide variety of applications. The findings indicate that SqueezeViT outperforms the current SOTA classifiers while maintaining a lightweight model architecture. In turn, such results emphasize the possibilities of using SqueezeViT in real clinical environment, where the amount of computational resources can be constrained.

## Introduction

The most recent developments in the field of deep learning methods have created a lot of academic interest due to their high performance in the context of medical image analysis, especially in terms of image classification and organ/lesion segmentation. Regarding the diagnosis of tuberculosis (TB), the presence of large, carefully annotated data sources has enabled the creation of supervised learning models that can automatically learn the salient features of imaging data. Empirical studies have shown that deep learning models are highly accurate and operationally efficient in detecting and classifying TB cases.

Considering the urgent need for rapid diagnostic approaches, especially in the case of a pandemic, automated detection of abnormalities on chest X-ray (CXR) images has become of paramount importance. The extensive use of digital technologies, such as mobile devices, digital sensors, and communication platforms, has driven rapid growth in structured, semi-structured, and unstructured data in medical care.

Recently, various techniques^1,3–5^ of automatic multi-label CXR classification has been introduced to enhance the accuracy, and can be broadly classified based on the methodology adopted, transfer learning based methods^1,3^, network innovation methods^6,7^, and attention-guided methods^5^. This digital expansion has motivated researchers to use deep learning methods, especially CNNs, for automated disease detection, identification, and classification from CXR images. Although these studies apply the CNN to obtain satisfactory classification, they still do not meet the critical requisites of medical image investigation. The primary issue is that early and superficial CNN-based algorithms are incapable of learning globally and capturing long-range semantic information in the convolutions^8^.

Inspired by the success of Transformers, which have the capability to learn global patterns, the researchers are inclined towards utilizing their strengths for complex medical image analysis. The ViTs^9^ have recently become the most suitable advanced technology for various complex vision tasks. However, the architectural complexity of ViTs and its large computational requirements pose a serious challenge, especially in resource-limited environments^10^. In order to address these shortcomings, hybrid models that combine convolutional neural networks (CNNs) and ViTs have been suggested, the aim of which is to combine the respective advantages of the two paradigms. However, the actual implementation of ViTs in low-resource settings in the real world is an imposing challenge.

The rest of this manuscript is structured in the following way: Sect. 2 is the literature review, and Sect. 3 outlines the datasets and the methodology. In Sect. 4, the experimental results along with the discussion of their implications are given. In addition, Sect. 5 presents an insight into ablation studies, and Sect. 6 is the conclusive statement.

## Related Work

Multi-class classification of CXR images is one of the most commonly adopted approaches in medical image analysis, and it is inseparable in the process of computer-aided diagnosis of the disease. Transfer learning has been used to solve this problem in some studies, specifically, Wang et al.^1^ have applied various classical convolutional neural network (CNN) models, such as AlexNet^11^, VGGNet^7^, GoogleNet^12^ and ResNet^12^ to identify the presence of several diseases in the ChestX-ray14 dataset. In contrast, Haritha et al.^2^ trained a slightly modified Densenet^13^ by replacing the last classifier layer for improved accuracy.

For mobile vision tasks, CNNs often offer high performance with less computational complexity as compared to transformers. Architectures like ResNet^7^ leverage residual connections to ease training, DenseNet^13^ promotes feature reuse through dense connectivity, and ShuffleNet^14^ reduces computational cost using group convolutions and channel shuffling. MobileNet introduces depthwise separable convolutions to achieve high efficiency, while SqueezeNet^15^ emphasizes reducing model size and latency through lightweight convolutional operations. These network architectures with fewer parameters and lower computational requirements, measured in multiply-accumulate (MAC) operations, are considered lightweight, making them easier to deploy^16^.

The performance of transformers in Natural Language Processing (NLP)^1^ inspired researchers^12,17–22^ to introduce transformers to Computer Vision (CV). Self-attention is also a crucial ingredient in the success of transformers, which is capable of capturing short-range and long-range visual relationships. ViT generalizes this architecture to handle vision tasks, where images are represented as sequences of non-overlapping patches^23^. They work just like tokens once you flatten them. The input feature map is reshaped as an LP sequence of patches that are embedded into vectors. These vectors are then self-attentively processed. Although ViT-style models have been shown to be effective in capturing global information and in many cases yield better performance compared to CNNs, they suffer from high computational demand, and the quadratic cost of self-attention calls for orders of magnitude more parameters.

Recent research suggests that combining CNNs with transformer architectures improves model efficiency while maintaining or surpassing existing performance benchmarks. Models like CoatNet^24^, CMT^25^, and BoTNet^26^ sequentially integrate CNN-based and transformer-based modules to enhance feature extraction and image understanding. TinyViT^27^ presents a compact ViT optimized for efficient image classification through knowledge transfer techniques and reduces the gap between lightweight and full-sized models. Mobile Former addresses mobile vision tasks by incorporating a lightweight, parallel transformer structure suitable for real-time applications. MobileViT further innovates by treating transformers as convolutions, restructuring patches to maintain the local pixel order, and removing the need for positional embeddings.

Recent developments in the field of CXR classification have taken advantage of the deep learning algorithm, whereby an emphasis has been made on transfer learning and transformer-based architectures to improve diagnostic performance. Pham and Hoang^28^ used the concept of transfer learning with hyperparameter optimisation to enhance the performance of pulmonary disease diagnosis, thus proving the effectiveness of fine-tuned pre-trained models in medical imaging. Similarly, Gu and Lee^29^ used deep transfer learning on real-world image data to screen against pneumonia and emphasized the importance of reusing features trained on large repositories of generic images in clinical imaging. Beyond the traditional CNN, in their work, Özturk et al.^6^ proposed a new multi-branch transformer-based model, HydraViT, that can be used to identify multiple disease patterns in multi-class CXR classification, and it demonstrated promising performance. Altogether, these research works demonstrate the development of a growing interest in hybrid CNN transformer models aimed at ensuring a compromise between the classification effectiveness and the computational cost of medical image analysis.

More recently, the NIH Chest X-ray collection^1^ and CheXpert^17^ have been used regularly as datasets in CXR classification studies. Table 1 lists the deep learning methods benchmarked for tuberculosis (TB) and chest disease detection using these CXR datasets. Competitive area under the curve (AUC) values were also achieved with models like BarlowTwins CXR^18^, which are based on ResNet-50 but with high computation requirements. Computational efficiency, which is often measured by operations like matrix multiplications, is a critical issue. Hybrid architectures using GoogleNet^12^ and ResNet backbones have alleviated TB classification performance but are still susceptible to detecting delicate abnormalities, thus underscoring the need to develop superior attention systems. Methods based on the Generalised Zero-Shot Learning (GZSL) were highly accurate but faced the challenge of generalisation. Attention Decoder Networks (ADNet)^19^ achieved good AUROC scores, but at an extra interpretability and execution cost. To address these challenges, the proposed methodology incorporates MobileViT to produce interpretable attention maps. Comparative studies have suggested that CNNs are highly accurate with quite low computational cost, but ViT is better at generalisation, at the cost of increased model complexity. The performance of the models is measured in terms of binary cross-entropy loss and AUC. Thus, this study strategically combines transformer-based attention mechanisms with a lightweight CNN to achieve an optimal trade-off between high performance and computational efficiency, which makes it appropriate to be deployed in clinical settings.

**Table 1:** Benchmarking Deep Learning Techniques for TB and Chest Disease Detection Using Chest X-rays Datasets.

Model	Methodology	Dataset	AUROC Score	AUROC (Local population)	AUROC (Demographic population)
(BarlowTwins-CXR, 2024)^18^	Self-supervised pre-training using the adjusted Barlow Twins algorithm with the ResNet50 backbone pre-trained on ImageNet	NIH Chest X-ray Dataset (112,120 images)	0.8107	✓ (Tested on NIH dataset)	✗ (Demographic-specific evaluation not performed)
(TB Classification using Custom CNN-ResNet, 2023)^20^	Custom model combining GoogLeNet (Inception) and ResNet architectures with additional layers	NIH Chest X-ray Dataset (for TB classification)	0.85	✓ (Trained and tested locally on NIH data)	✗ (No demographic analysis)
(Multi-Label Generalized Zero-Shot Chest X-ray Classification, 2025)^21^	Generalized Zero-Shot Learning combining image-text feature disentanglement	Chest X-ray Dataset (unspecified)	0.8830	✓ (Trained and tested on available datasets)	✗ (Model bias toward seen classes noted)
(Attentional Decoder Networks (ADNet), 2024)^19^	Encoder–decoder attentional decoder network using high-resolution feature maps	Chest X-ray Dataset (unspecified)	0.853	✓ (Focused on local features)	✗ (Demographic-specific accuracy not evaluated)
(Comparative Study of CNN, ResNet, Vision Transformers, 2024)^22^	CNNs, Residual Networks (ResNet), Vision Transformers comparison study	Chest X-ray Datasets (likely NIH, others unspecified)	0.86	✓ (Focused model optimization)	✗ (Demographic differences not evaluated)
(Interpretability Techniques for Deep COVID-19 Classification, 2020)^30^	Ensemble models (DenseNet161, InceptionResNetV2, InceptionV3, ResNet18, ResNet34)	COVID-19 Chest X-ray Dataset (unspecified)	0.8510	✓ (Dataset-specific results)	✗ (Demographic-specific analysis not conducted)
(MLVICX: Multi-Level Variance-Covariance Exploration, 2024)^31^	MLVICX: Multi-Level Variance-Covariance Self-Supervised Representation Learning	Chest X-ray Dataset (unspecified)	0.8610	✓ (Trained on available datasets)	✗ (Demographic-specific generalization not tested)

Powerful classifiers such as DenseNet-121, GoogleNet, and ResNet were initially implemented to classify natural images. However, the networks have been trained on extremely large datasets exceeding one million images and distinguishing between thousands of classes, thus requiring substantial memory and computing resources for both training and inference.

The main goal of this research is to design and develop a lightweight Vision ViT model, named SqueezeViT, that effectively classifies chest X-ray (CXR) images. The proposed model is a simple approach for CXR diagnosis, introducing a token-squeezing system that reduces the number of computations required, while preserving key diagnostic information in the images. This proposed model is a computationally efficient architecture that delivers robust, generalizable performance. It combines the advantages of MobileViT and the SqueezeNet, which are efficient models that are based on the ViT and CNN families, respectively. The MobileViT block forms the basis of using transformers to approximate convolutional operations; this transformer keeps the global information, but the local pixel order is preserved. SqueezeNet Fire module aids in reducing the parameters.

The design methodology focuses on retaining larger feature maps in the network. The model consists of two critical blocks; the Fire block, inspired by the Fire module to learn efficiently local representation, and the Translution Block, which is a variant of the MobileViT block that is adapted to learn efficiently global contexts. This combination facilitates effective feature learning, and the total number of parameters is reduced.

The suggested model is tested on two publicly available CXR classification datasets, namely NIH ChestX-ray14 and CheXpert, to assess its performance. Extensive experiments were conducted using SqueezeViT’s performance metrics, with the outcomes compared to state-of-the-art (SOTA) methods, thereby demonstrating the feasibility of applying SqueezeViT in clinical practice, especially in low-resource healthcare environments.

The following are the objectives of this research:

- Design an efficient resource-conscious ViT, SqueezeViT, to optimize the classification of CXR images.
- Design a token-squeezing algorithm that allows reducing costs in computations but still retains important diagnostic features of medical images.
- Test the performance of the designed model for CXR benchmark datasets, NIH ChestX-ray14, and CheXpert.
- Compare and contrast the classification accuracy and efficiency of SqueezeViT with other deep-learning models, such as CNN and ViT-based architectures.
- Show that SqueezeViT can be implemented in environments of realistic feasibility, specifically in low-resource healthcare settings.

## **Dataset and Methodology**

In this section, the dataset and the proposed methodology have been described in detail. The publicly available databases, such as the NIH Chest X-ray dataset and CheXpert, were used in terms of exploring the classification performance. The dataset was normalized and rescaled to 224 × 224 single-channel grayscale images, then split randomly into an 80% training set and a 20% testing set. As evaluation metrics, class-wise and average AUROC were used in the multi-label classification framework. In binary classification problems, the main performance measure was AUROC.

### Dataset I

NIH Chest X-ray Dataset^1^.

**Fig. 1:**
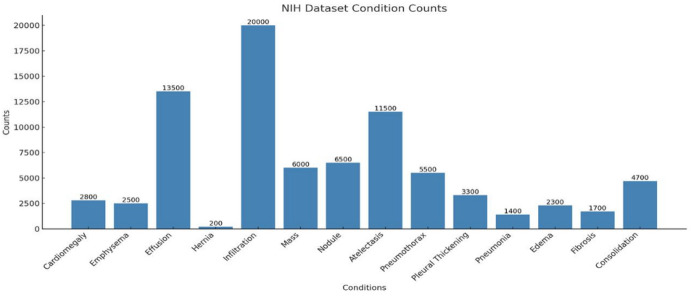
Distribution of disease conditions in the NIH Dataset, showing the frequency counts for various medical conditions identified from X-Ray images.

The aim of the current experimental setup is to compare chest radiograph images using multilabel and binary classification. The dataset also includes 112,120 chest radiographs from 30,805 patients and annotations for 14 diseases, such as pneumonia, atelectasis, cardiomegaly, and effusion; this is a complete resource for multi-disease classification. The distribution of each class in the NIH Chest X-ray dataset is detailed in Fig. 1.

### Dataset II

CheXpert Dataset^17^.

**Fig. 2:**
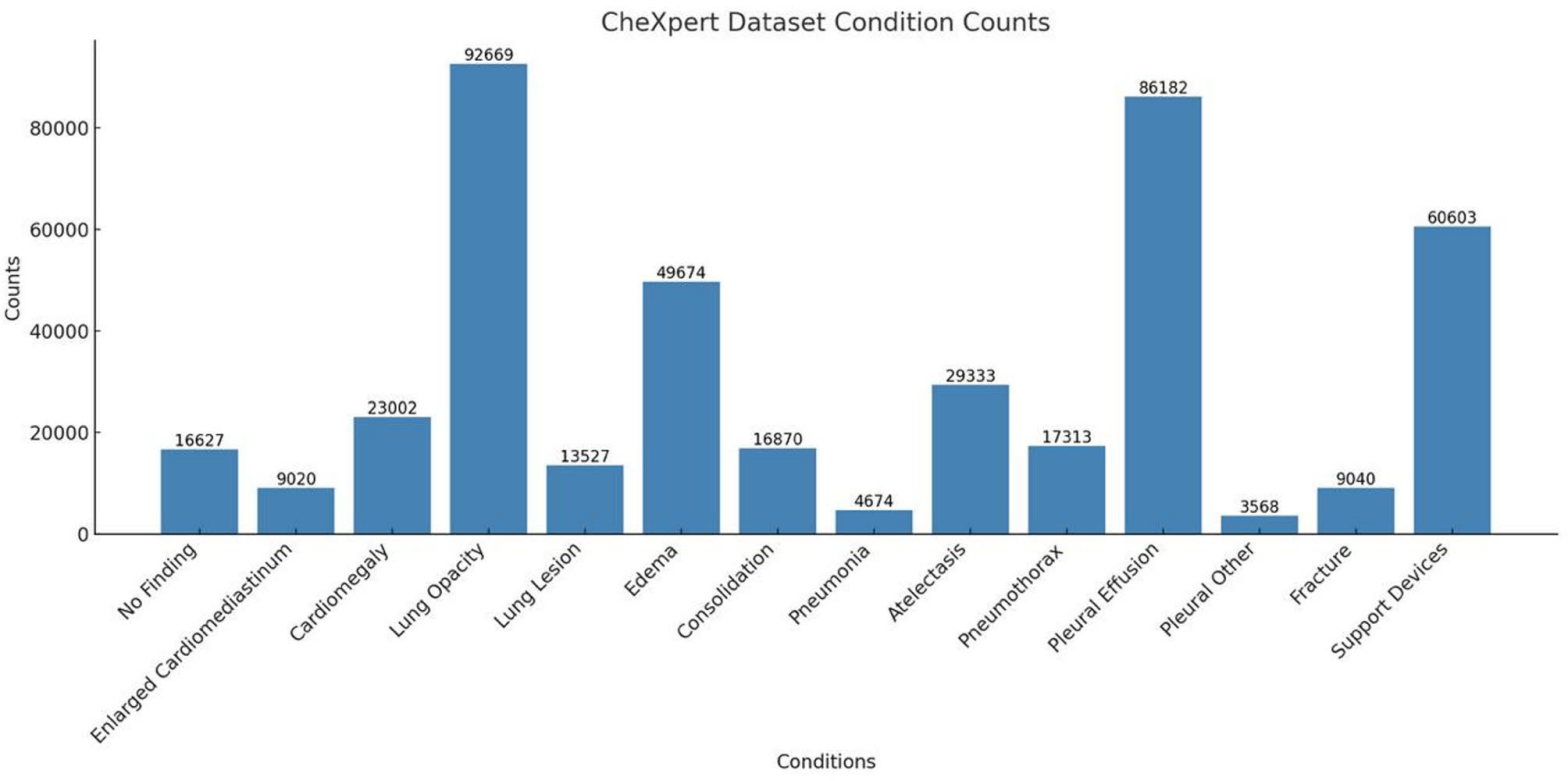
Distribution of disease conditions in the CheXpert Dataset, showing the frequency counts for various medical conditions identified from X-Ray images.

The CheXpert dataset is a huge dataset for automated CXR diagnosis and is commonly used in medical imaging research. It contains 224,316 images from 65,240 patients, in both frontal and lateral projections. The specifics of each class in the utilized CheXpert dataset are illustrated in Fig. 2. Each image is annotated for the presence of 14 different observations as listed in chest radiographs, including atelectasis, cardiomegaly, consolidation, edema, and pleural effusion.

### SqueezeViT

**Fig. 3:**
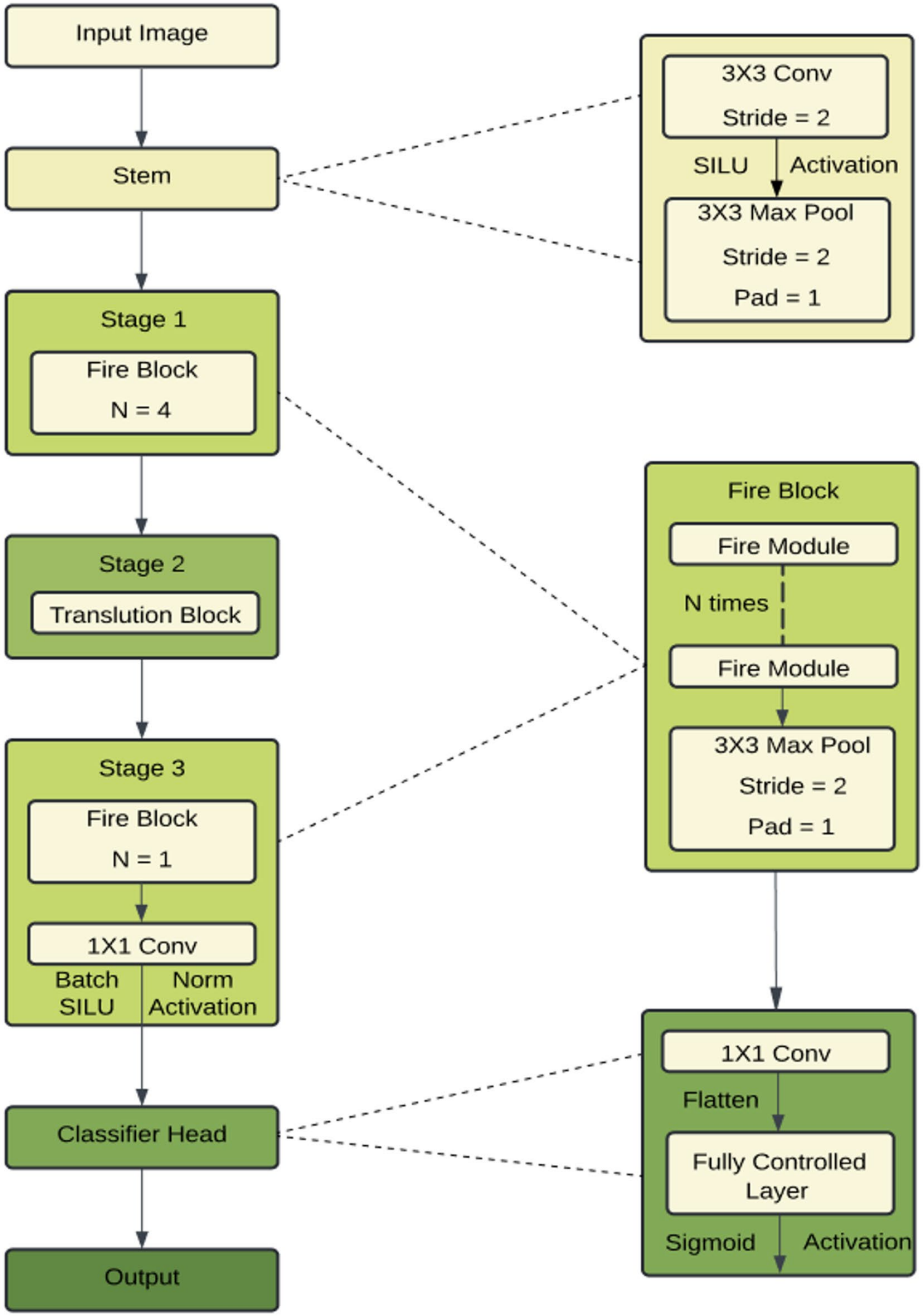
Schematic architecture of the proposed SqueezeViT model.

**Fig. 4:**
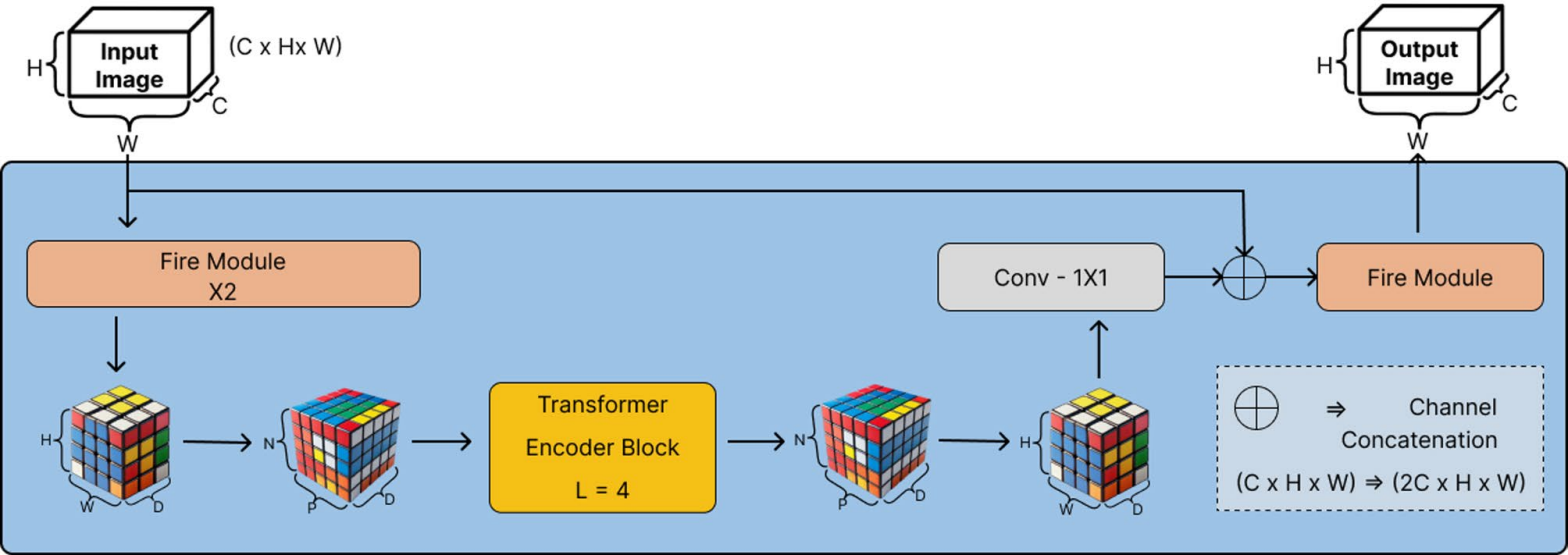
Schematic of Translution Block with key parameters and its settings.

The general network architecture of the presented model, SqueezeViT, is depicted in Fig. 3, whereas Fig. 4 describes the important architectural parameters and settings for the Translution Block in stage two of the network. SqueezeNet^15^ employs a Fire module to minimize model parameters. The proposed design heavily takes inspiration from SqueezeNet. The model we propose, SqueezeViT, incorporates Fire modules along with intentional downsampling of feature maps to maintain larger feature sizes. Additionally, we present the Translution Block, an architectural adjustment to the MobileViT block, aimed at further decreasing computations. The combination of learning local context through Fire modules and global contexts through the Translution Block is crucial in developing a model that is generalizable, straightforward, and computationally efficient.

The SqueezeViT network is comprised of a stem followed by three different stages, as depicted in Fig. 3. The stem consists of a $\:3\times\:3\:$convolutional layer, having three output channels, and a max-pooling layer. This initial stage effectively reduces the feature map size early on to lower computational cost while extracting features.

For an input of dimension $\:({C}_{in}\:\times\:\:H\:\times\:\:W)$, the convolutional operation adjusts it to $\:{C}_{out\:}\times\:\:H{\prime\:}\:\times\:\:W{\prime\:}$.

where $\:H{\prime\:}\:=\:[\frac{H\:+\:2p\:-\:k}{s}\:+\:1]$ and $\:W{\prime\:}\:=\:[\frac{W\:+\:2p\:-\:k}{s}\:+\:1]$, with $\:k=\:kernel\:size$, $\:s\:=\:stride$ and $\:p\:=\:padding$.

Stage 1 involves the use of Fire Block to create localized representations in an efficient way. This step is completed by a max pooling layer that downsizes the feature map. The down-sampling that is carried out at the end of the stage is retained as a comparatively larger feature map, which alleviates the computational requirements of the following stages.

Stage 2 adds a translational block that is useful in capturing the global context without altering the local pixel structure and resolution of the feature map.

Stage 3 includes a Fire Block followed by max pooling to obtain both the global and local framework while simultaneously reducing computation. Relying solely on global pooling could lead to a loss of spatial information, thus motivating the use of a 1 × 1 convolution before global average pooling.

The global average pooling operation produces an output of shape: $\:\left(C,\:1,\:1\right)\:\:\:$where $\:C$ is the number of channels. After flattening, this leads to an input vector for the fully connected layer used for multi-label classification.

### Fire block

We utilize a series of Fire modules to form our Fire Block, which concludes with a 3 × 3 max pooling layer to down sample the generated feature map, as shown in Fig. 4. A Fire module processes the input feature map $\:X$ through two key stages is shown in Eq. 1.

Squeeze step:

1 $$\:\begin{array}{c}{X}^{{\prime\:}}=ReLU\left({W}_{s}\:*\:X+{b}_{s}\right)where\:{W}_{s}\:\in\:\:{R}^{{C}_{s}\:\times\:\:{C}_{in}}\end{array}$$

Here $\:{W}_{s}\:$and $\:{b}_{s}$ are learnable weight and bias parameters that project the original $\:{C}_{in}$channels down to a reduced $\:{C}_{s}$, and is ReLU activation, which brings non-linearity and keeps it sparse. The reduction of channels decreases the number of tokens input to the subsequent ViT module, thereby directly diminishing its attention-related FLOPS. Additionally, it allows the network to function as an information bottleneck, which further incentivizes the network to retain only the most salient features. This results $\:{C}_{s}\:<<\:{C}_{in}\:$in an empirical gain in the effective inference speed, and only modestly affects downstream classification accuracy.

Excitation step $\:{e}_{1\:\times\:\:1}\:$ and $\:{e}_{3\:\times\:\:3}$ is shown in Eq. 2

2 $$\:\begin{array}{c}{e}_{1\:\times\:\:1}=ReLU\left({W}_{1\:\times\:\:1\:}*\:{X}^{{\prime\:}}+{b}_{1\:\times\:\:1}\right)and\:{e}_{3\:\times\:\:3}=ReLU\left({W}_{1\:\times\:\:1\:}*\:{X}^{{\prime\:}}+{b}_{3\:\times\:\:3}\:\right)\end{array}$$

where $\:{W}_{1\:\times\:\:1}$​ and $\:{W}_{3\:\times\:\:3}\:$are the weights of the parallel 1 × 1 and 3 × 3 convolutional filters, respectively, and $\:{b}_{1\:\times\:\:1}\:$ and $\:{b}_{3\:\times\:\:3}$ are the corresponding bias terms.

The final output $\:{F}_{out\:}\:$of a Fire module is obtained by concatenating the outputs of both excitation branches along the channel dimension, as shown in Eq. 3

3 $$\:\begin{array}{c}{F}_{out\:}=\:Concat\left({e}_{1\:\times\:\:1},\:{e}_{3\:\times\:\:3}\right)\end{array}$$

where $\:*$ denotes convolution, and $\:Concat$ denotes channel concatenation.

To ensure homogeneity across feature maps, the squeeze layer $\:{s}_{1\:\times\:\:1}$​ outputs half the number of input channels for its respective Fire Block. Each excitation layer $\:{e}_{1\:\times\:\:1}$ and $\:{e}_{3\:\times\:\:3}\:$individually produces half of the final output channels.

In Stage 1, the Fire Block consists of $\:N\:=\:4$ Fire modules. The channel progression can be described as:

$$\:3\to\:32\to\:64\to\:64\to\:128$$

Fire modules are more computationally efficient compared to conventional convolutional layers, significantly decreasing the computational drain even with a higher count of the feature map. As a result, local representations can be effectively learned without requiring extensive computations.

In Stage 3, a single Fire module increases channels from: $\:128\to\:256$. This expansion enables the acquisition of local and global contextual knowledge acquired at the stages 1 and 2 thus enhancing the feature representation of the network at later stages.

### Translution Block

The second stage of the proposed architecture, the Translution Block, depicted in Fig. 4, is meant to facilitate stronger learning of global representation. In this block, $\:H$ and $\:W$ represent the height and width of the entire input feature map, while $\:h$ and $\:w$ represent the height and width of individual image patches obtained from the entire image feature map. The full feature map of size $\:H\:\times\:\:W$ is partitioned into smaller patches of size $\:h\:\times\:\:w$, where the patch size is denoted by: $\:\:\:\:\:\:P\:=\:h\:\times\:\:w$ and the total count of patches is given by: $\:N\:=\:\frac{H\:\times\:\:W}{h\:\times\:\:w}$.

Following the architectural design principles adapted from the MobileViT block, the input feature map of resolution $\:(H\:\times\:W)$ with $\:C$ channels are first processed through two sequential Fire modules. The first Fire module increases the number of channels from 128 to 256, while the second Fire module reduces it from 256 to 64, thereby enhancing the depth and width of the network for more effective local representation learning compared to the original design.

We reshape the down-sampled feature maps provided by the SqueezeNet backbone into temporally ordered flat tokens (tokens) and project them into query, key, and value spaces of dimension $\:{d}_{k}$(for queries and keys) and $\:{d}_{v}$​ (for values). Finally, the self-attention between these two tokens is calculated described in Eq. 4

4 $$\:\begin{array}{c}Attention\left(Q,\:K,\:V\right)=\:softmax\left(\frac{Q{K}^{T}}{{d}_{K}}\right)V\end{array}$$

Where, $\:Q,K\in\:{R}^{N\times\:{d}_{k}}$ and $\:V\in\:\:{R}^{N\times\:{d}_{v}}$, with $\:N$ equal to the number of tokens. The division by $\:\sqrt{{d}_{k}}$ counteracts the tendency of large dot-product values to push the softmax into regions with extremely small gradients, thereby stabilizing training.

The relevance of each value vector $\:{V}_{j}$ to query $\:{Q}_{i}$ allows the model to combine global context at all spatial locations in a single, fully differentiable step, a property that is needed to capture long-range dependencies but does not require the parameter overhead of a pure CNN. The transformer Encoder carries out the acquisition of global representations by taking pixel-wise interaction between patches. Patches of size $\:(4\:\times\:\:4)$ are chosen in order to optimize computational efficiency without excessive local context. This option minimizes the patches and the general complexity of the computation, in contrast to the smaller patches of $\:(2\:\times\:\:2)$. In particular, this reduction can be obtained by increasing the patch size from $\:\left(2\:\times\:\:2\right)$ to $\:(4\:\times\:\:4)$, as given in Eq. 5.

5 $$\:\begin{array}{c}{N}_{4\:\times\:\:4}\:=\:\frac{{N}_{2\:\times\:\:2}}{4}\:\:and\:\:\:\:FLOP{s}_{4\:\times\:\:4}\:=\:\frac{FLOP{s}_{2\:\times\:\:2}}{4}\end{array}$$

Since each attention head processes one token per patch, using $\:(4\:\times\:4)$ patches reduce both the token count and the per-layer FLOPS by a factor of four. Empirically, this patch-size choice yields nearly the same classification accuracy on benchmark datasets, while cutting the ViT module’s runtime roughly in half compared to the finer $\:(2\:\times\:2)$ partitioning—thereby meeting our goal of time-efficient inference with minimal degradation in performance. After transformer-based features are merged back with the original input features through a 1 × 1 convolution, followed by channel-wise concatenation, effectively doubling the channel dimension as,

$$\:{C}_{output}\:=\:2C$$

Finally, another Fire module efficiently reduces the channel count back from $\:2C\underrightarrow{FIRE\:BLOCK}C$. Thus, preserving the learned representations while maintaining a lightweight architecture.

Overall, the Translution Block uses transformer-based attention mechanisms to obtain global context learning and Fire modules to obtain efficient local representations and dimensionality reduction. The suggested architectural changes, including increased patch size, the sequential rollout of Fire modules, and an optimized transformer encoder, yield the optimum Translution Block.

The AUROC has been considered a useful metric for assessing the performance of classification models, particularly in complex settings, for multi-label classification. The ratio of true positives to the sum of true positives and false negatives at all thresholds provides a holistic indication of discriminative ability, known as AUROC. In contrast to accuracy, which can be vulnerable to this distortion in cases of class imbalance or when cases cross multiple classes, AUROC is robust because it focuses on the relative rankings of true positives versus false positives. Moreover, class-wise AUC scores ensure that performance is evaluated independently for each class, preventing dominant classes from overshadowing the behaviour across less frequent labels. This makes AUC a more reliable and nuanced metric for assessing model performance in imbalanced and multi-class settings.

## Results and discussion

In this section, we evaluate the efficiency of SqueezeVit and also compare SqueezeVit with the SOTA methods to show the superiority of our method. Our experimental design is focused on the tasks related to multilabel and binary classification by CXR diagnosis. The study uses two separate datasets for this purpose and compares the proposed methodology with several SOTA techniques available in the literature. This includes computationally expensive models such as ResNet-18^7^ and DenseNet 121^13^, and lightweight models such as SqueezeNet^15^, ShuffleNetv2^32^, MobileNetv2^33^, and also ViTs like MobileViT^34^. Therefore, our comparative study provides an in-depth analysis of the performance of the designed SqueezeViT model.

For the experiment, we set a batch size of 64 and 100 training epochs. All models are trained to minimize binary cross-entropy loss using the Adam optimizer. The learning rate is set to $\:1{e}^{-4}$. For multilabel classification problems, we use class-wise AUROC and average AUROC as performance metrics. For binary classification, we use AUROC as our primary performance metric. The hyperparameters of the proposed SqueezeViT model were selected through systematic empirical experimentation and insights from prior work on tuning ViT for medical image analysis^35^. The models are trained with the images resized to 224 × 224 pixels. Each model is trained from scratch in the experiments. Robustness of the model is ensured through multiple training iterations, with the model demonstrating consistent convergence.

### Computational complexity testing

The proposed SqueezeVit model exhibits exceptional performance across all key metrics, i.e., parameters (∼10⁶), MACs (∼10⁹), training time (in hours), GPU usage (in GB), CPU latency (in ms), and throughput, indicating the number of images processed per second. The results achieved by the suggested model prove to be a viable benefit when it comes to the actual deployment in the real world. The proposed model has a lower parameter count of $\:0.54\: \times \:\:{10^6}$, making it a lighter model that uses less memory and has a higher loading efficiency. The MAC operations are $\:0.37\:\times\:{10}^{10}$, which shows lower arithmetic complexity, allowing faster inference and lower power consumption. The training time is 3.5 h; reduced training time accelerates both experimentation and model inference, while reducing computational cost. The model uses 1.2 GB of GPU memory, enabling it to run on devices with limited resources. Lastly, the CPU latency is 6ms, which allows it to respond quickly when making an inference, an attribute essential for real-time applications. All these factors together substantiate the scalability, affordability, and feasibility of the proposed approach for a real-time and patient-independent diagnosis system. Table 2 compares the computational complexity of the SqueezeVit model with other models, clearly demonstrating its computational efficiency.

**Table 2:** Model comparison based on Parameters, Computation, GPU Usage, Latency, and Training Time.

Model	Params (10^6)	MAC (10^9)	Training Time (hrs)	GPU Usage (GB)	CPU Latency (ms)	GPU Latency (ms)	Throughput (img/s)
**ResNet**	11.69	1.81	13.5	7.5	27.5	6.8	147
**DenseNet**	7.98	2.83	15.5	9	37.5	8.9	112
**SqueezeNet**	1.25	0.83	2.5	0.85	4	1.9	526
**MobileNet**	3.5	0.3	4.5	1.3	6.5	2.6	384
**ShuffleNet**	7.39	0.58	3.5	1	5	2.3	435
**MobileViT**	0.95	0.2	6.5	2.5	11	3.5	285
**SqueezeVit**	0.54	0.37	3.5	1.2	6	2.1	476

### Multilabel Classification

To compare the performance, we calculate AUROC averages for the different anomalies for each dataset. These results are presented in Table 3 for the NIH dataset and Table 4 for the CheXpert dataset. Our comparisons include not only computationally inexpensive models such as SqueezeNet, MobileNet, and ShuffleNet, but also the heavy models such as ResNet and DenseNet. It is noteworthy that the proposed SqueezeVit model clearly outperforms other lightweight models in the vast majority. Furthermore, while DenseNet and ResNet are computationally intensive, they yield results comparable to those of the proposed lightweight model. The results show that the proposed model has an optimal balance between predictive performance and computational complexity.

In evaluating multilabel classification across two diverse datasets using various models, distinct patterns emerged. For the NIH dataset, as shown in Table 3, models such as ResNet, DenseNet, and the proposed SqueezeViT demonstrated prominently strong performance for most labels, frequently achieving AUROC scores above 0.7. Conditions like Cardiomegaly and Edema were detected effectively, whereas labels such as Nodule and Pneumothorax proved more challenging. Regarding the Chexpert dataset, showed in Table 4, ResNet and DenseNet consistently performed well, recording AUROC scores primarily above 0.7, while labels like No Finding and Pleural Effusion achieved higher AUROC scores.

Even though the suggested SqueezeViT provides good results with reduced computational complexity compared to other models, the loss of spatial information associated with the patch tokenization procedure may lead to prediction errors, and incorrect attention alignment may result in missed edges and textures. Table 5 shows chest X-ray samples from the NIH dataset, comparing the actual diagnosis to the model’s prediction. It is observed that diseases such as Enlarged Cardiac Mediastinum and Atelectasis are difficult to categorize due to their low contrast and soft boundaries. The proposed methodology can be further improved by using data-efficient training regimes and augmenting the training data to attain class balance.

**Table 3:** AUC’s for various abnormalities using different methods when trained and tested on the NIH dataset.

Label	ResNet	DenseNet	SqueezeNet	MobileNet	ShuffleNet	MobileViT	SqueezeVit (proposed model)
Atelectasis	0.713	**0.723**	0.709	0.683	0.691	0.679	**0.723**
Cardiomegaly	**0.862**	0.86	0.857	0.812	0.815	0.789	0.834
Effusion	**0.786**	0.786	0.78	0.751	0.746	0.75	0.784
Infiltration	0.671	0.661	0.671	0.654	0.644	0.648	**0.672**
Mass	0.700	**0.723**	0.717	0.636	0.616	0.627	0.696
Nodule	0.625	0.655	0.631	0.576	0.577	0.599	**0.656**
Pneumonia	**0.655**	0.652	0.638	0.636	0.611	0.613	**0.655**
Pneumothorax	0.763	0.788	0.771	0.742	0.750	0.735	**0.782**
Consolidation	**0.718**	0.713	0.707	0.700	0.695	0.696	0.708
Edema	**0.824**	0.819	0.819	0.799	0.796	0.785	0.816
Emphysema	**0.745**	0.742	0.725	0.685	0.686	0.675	0.732
Fibrosis	**0.76**	0.735	0.732	0.721	0.722	0.695	0.741
Pleural Thickening	**0.718**	0.714	0.704	0.691	0.673	0.678	0.708
Hernia	**0.851**	0.822	0.793	0.775	0.81	0.721	0.783

**Table 4:** AUC’s for various abnormalities using different methods when trained and tested on the CheXpert dataset.

Label	ResNet	DenseNet	SqueezeNet	MobileNet	ShuffleNet	MobileViT	SqueezeVit (proposed model)
No Finding	0.860	0.865	0.860	0.852	0.848	0.856	**0.861**
Enlarged Cardiomediastinum	0.633	0.653	0.647	0.637	0.633	0.629	**0.646**
Cardiomegaly	0.837	**0.847**	0.842	0.829	0.826	0.828	0.824
Lung Opacity	0.705	**0.709**	0.703	0.700	0.694	0.705	**0.709**
Lung Lesion	0.722	**0.741**	0.728	0.706	0.711	0.709	0.719
Edema	0.817	**0.832**	0.826	0.813	0.812	0.816	0.819
Consolidation	0.692	**0.712**	0.702	0.685	0.683	0.692	0.691
Pneumonia	0.688	**0.710**	0.693	0.661	0.655	0.677	0.689
Atelectasis	0.662	**0.673**	0.667	0.657	0.656	0.657	**0.673**
Pneumothorax	0.786	**0.820**	0.809	0.774	0.766	0.780	0.782
Pleural Effusion	0.852	0.867	0.858	0.839	0.830	0.849	**0.869**
Pleural Other	0.772	**0.790**	0.774	0.756	0.744	0.752	0.768
Fracture	0.686	**0.722**	0.710	0.702	0.685	0.694	0.700
Support Devices	0.804	**0.832**	0.819	0.794	0.779	0.811	0.797

**Table 5:** Sample chest X-ray images from the NIH dataset illustrating ground truth diagnoses versus model predictions. Correct predictions are indicated in green, while incorrect predictions are highlighted in red.

Image Examples						
Ground Truth	Consolidation, Fibrosis	Pneumothorax, Emphysema	Nodule, Edema	No Finding	Effusion, Cardiomegaly	Consolidation, Infiltration, Pneumonia
Predicted	**Consolidation**, **Fibrosis**	Pneumothorax	**Nodule**, **Edema**	**No Finding**	**Effusion**, **Cardiomegaly**	Emphysema, Infiltration

**Fig. 5:**
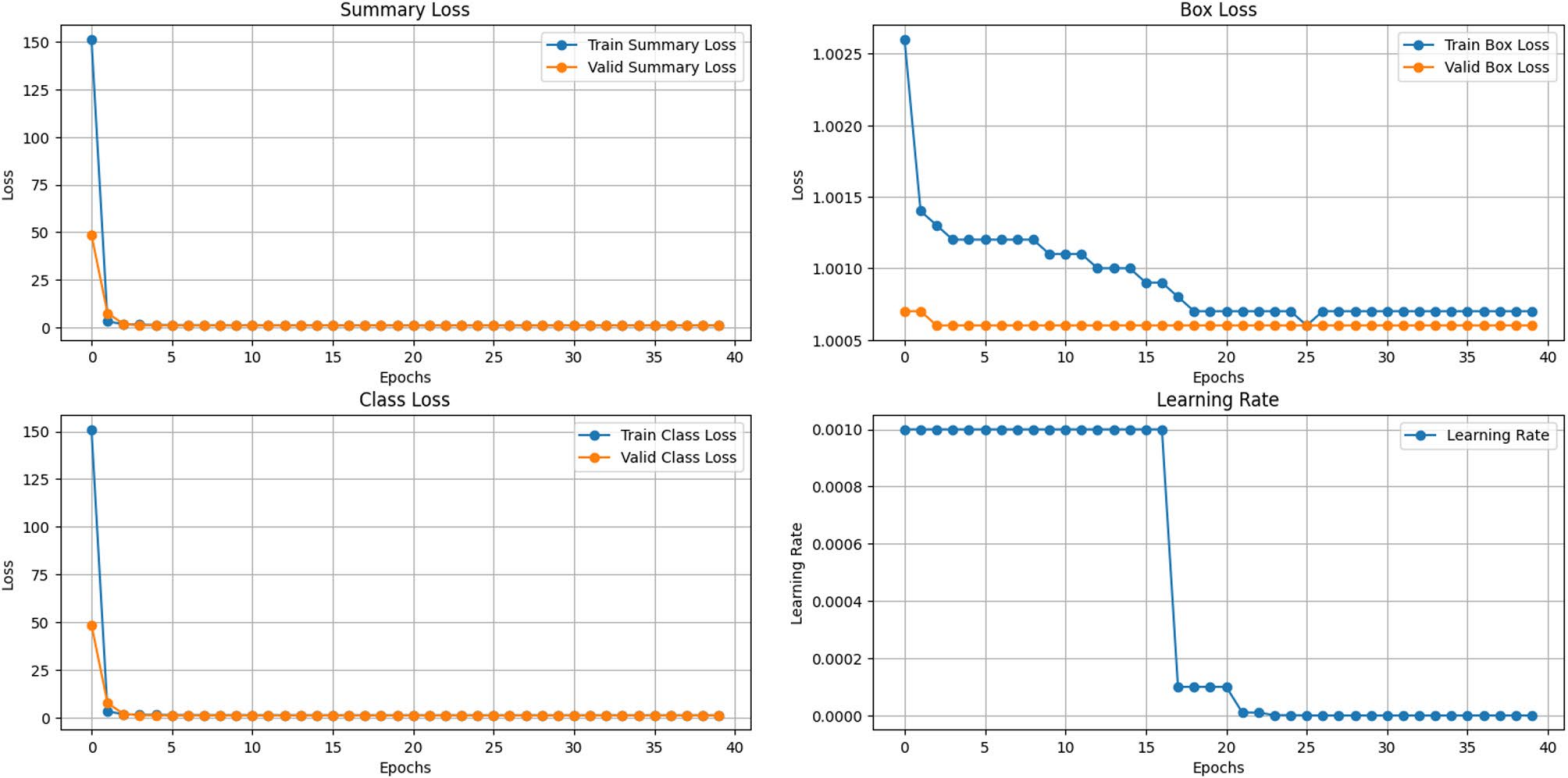
Visualization of Summary Loss, Box Loss, Class Loss, and Learning Rate Dynamics, during Training.

**Fig. 6:**
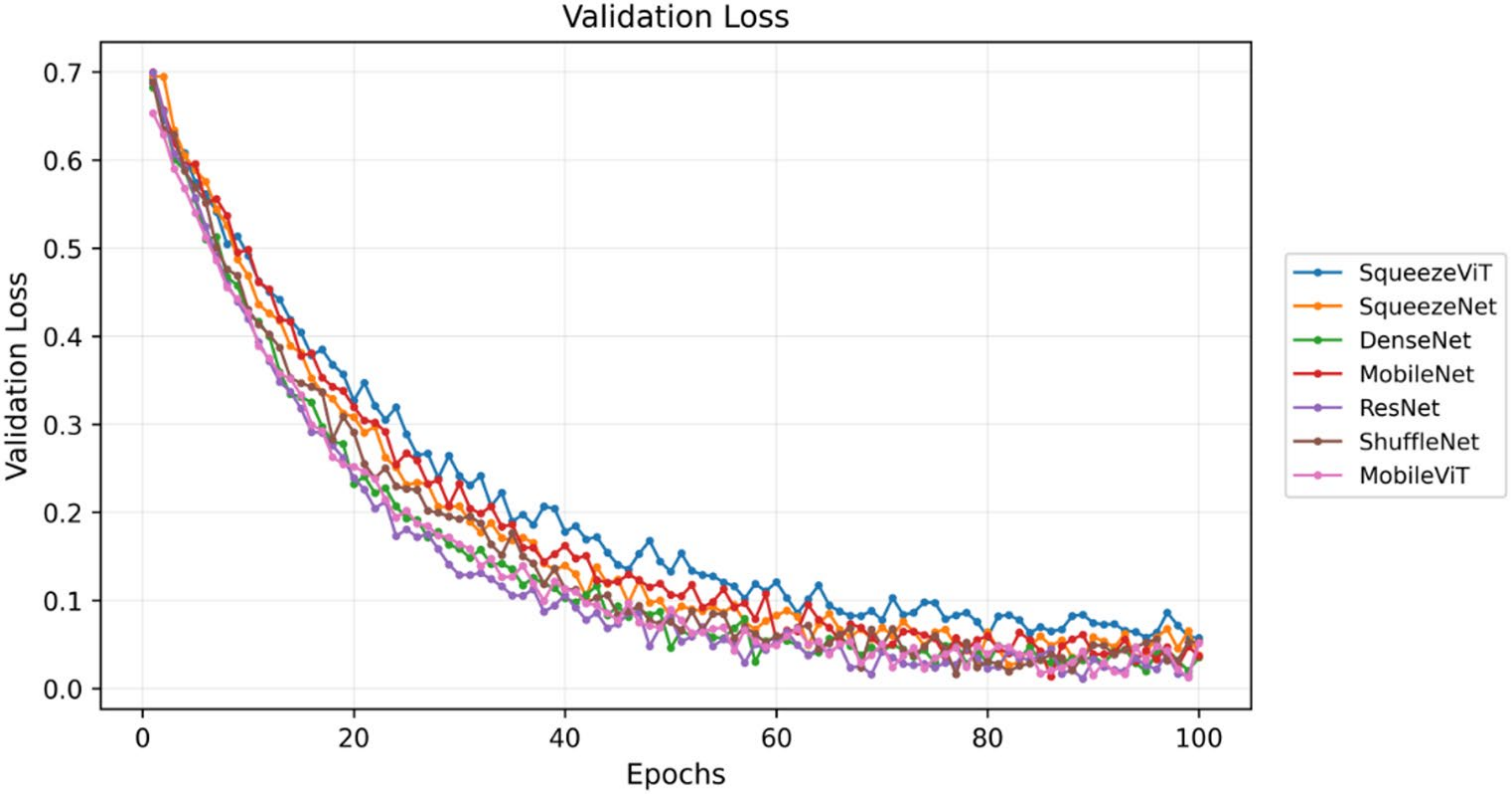
Validation loss curves across 100 epochs for various neural network architectures, illustrating their convergence behaviours and relative stability during model training.

**Fig. 7:**
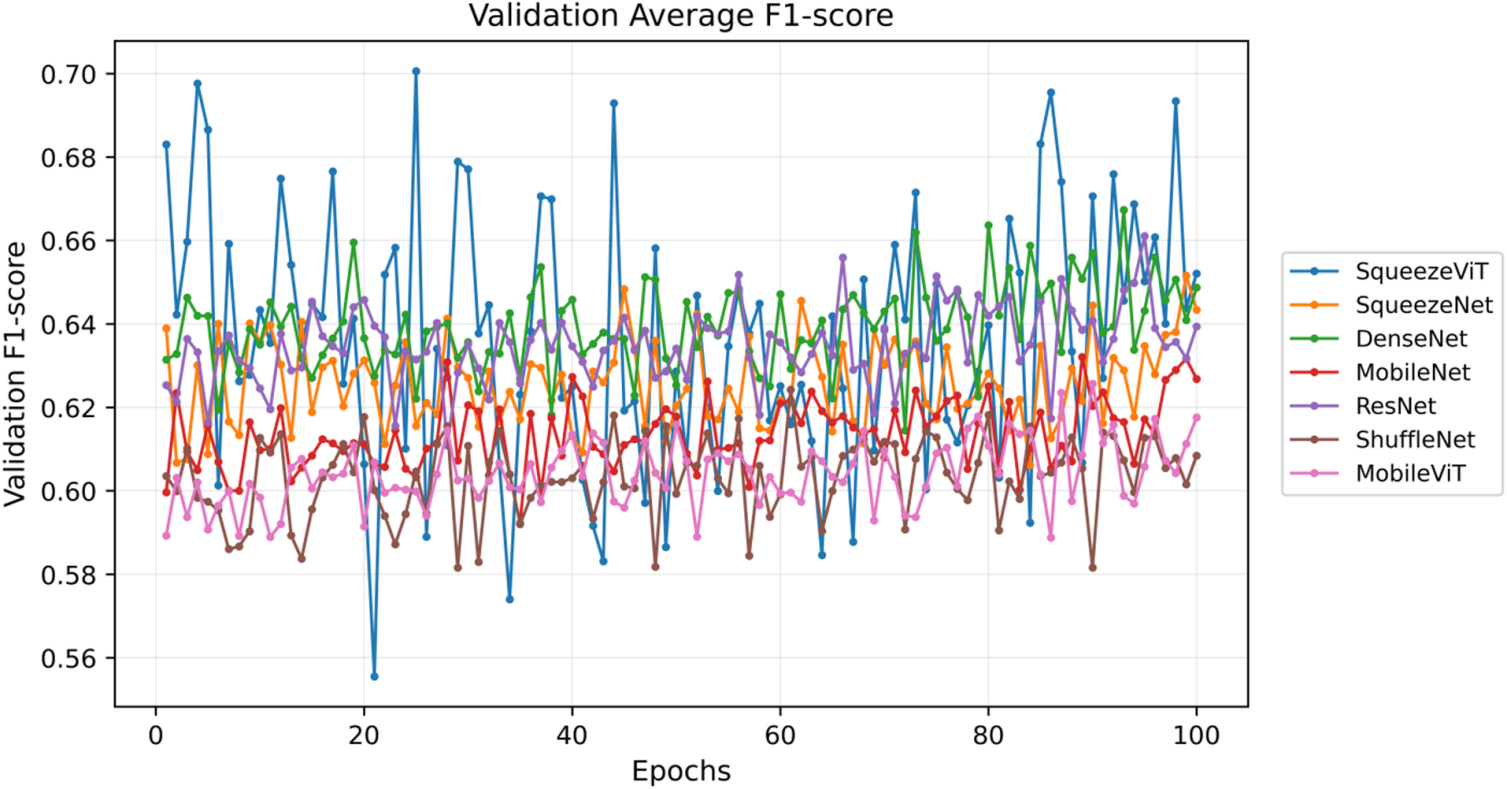
Validation average F1 scores over 100 epochs for various neural network models, illustrating their relative performance stability and classification accuracy throughout training.

The findings are presented in Figs. 5, 6 and 7 and provide a summary of the model’s performance and behaviour during training. Figure 5 illustrates the temporal dynamics of the summary loss, box loss, class loss, and learning rate, thus explaining the dynamics of learning behaviour of the model over time. Figure 6 shows the learning curves of different models for 100 validation epochs, from which significant differences in convergence speed and training stability could be observed. Simultaneously, Fig. 7 shows the F1 score and its mean for the models across 100 epochs, which provides information about their accuracy and consistency. All these findings testify to the stability and high performance of the proposed method in terms of training.

### Statistical significance testing of SqueezeVit

In order to determine the statistical significance of the proposed model performance, 95% confidence intervals (CI) of the values of the AUC of each class are provided in Table 6 for the case of the NIH dataset. Moreover, the p-values are also represented to give a comparison of the best baseline model with the p-values.

**Table 6:** Statistical Significance Analysis of AUC on NIH Dataset.

Abnormality	Best Baseline (AUC)	SqueezeViT AUC	95% CI	ΔAUC	*p*-value
Atelectasis	DenseNet (0.723)	0.722	[0.717, 0.727]	−0.001	0.42
Cardiomegaly	ResNet (0.862)	0.834	[0.829, 0.839]	−0.028	< 0.01
Effusion	DenseNet (0.786)	0.784	[0.779, 0.789]	−0.002	0.36
Infiltration	ResNet (0.672)	0.670	[0.665, 0.675]	−0.002	0.31
Mass	DenseNet (0.723)	0.696	[0.691, 0.701]	−0.027	< 0.01
Nodule	DenseNet (0.655)	0.646	[0.641, 0.651]	−0.009	0.03
Pneumonia	ResNet (0.656)	0.655	[0.650, 0.660]	−0.001	0.48
Pneumothorax	DenseNet (0.788)	0.782	[0.777, 0.787]	−0.006	0.04
Consolidation	ResNet (0.718)	0.708	[0.703, 0.713]	−0.010	0.02
Edema	ResNet (0.824)	0.816	[0.811, 0.821]	−0.008	0.03
Emphysema	ResNet (0.745)	0.732	[0.727, 0.737]	−0.013	< 0.01
Fibrosis	ResNet (0.760)	0.741	[0.736, 0.746]	−0.019	< 0.01
Pleural Thickening	ResNet (0.718)	0.708	[0.703, 0.713]	−0.010	0.02
Hernia	ResNet (0.851)	0.783	[0.778, 0.788]	−0.068	< 0.001

The statistical significance analysis reveals that the proposed SqueezeViT model is able to provide competitive, reliable diagnostic performance in comparison to the strongest CNN baselines. The small 95% confidence intervals of all the abnormalities indicate consistency and stability of the AUC estimates, which are driven by the large number of cases. In various diseases, such as Atelectasis, Effusion, Infiltration, and Pneumonia, the performance difference between SqueezeViT and the best baseline models is statistically insignificant (*p* > 0.05), indicating that SqueezeViT performs comparably to the best baseline models. Even though statistically significant differences are found in some of the abnormalities, including Cardiomegaly, Mass, Fibrosis, and Hernia, the overall findings justify the robustness of the suggested method and its generalization potential, while identifying the specific categories that can be enhanced in the future.

### Performance of SqueezeVit for Binary Classification

In this subsection, the effectiveness of the proposed model is compared to other competing models in classifying CXR as normal or abnormal. In this regard, we classify all CXR images that have been labeled as No Finding as normal, with the rest of the images being labeled as abnormal. Table 7 illustrates the results of binary classification, which show the AUROC. Based on the findings, one can conclude that the proposed model is superior to other lightweight models. Additionally, the proposed SqueezeVit model exhibits a performance that is either comparable to or surpasses that of the heavyweight models.

**Table 7:** Average AUROC for Binary classification of CXR using different methods.

Model	NIH	CheXpert
ResNet	0.688	0.849
DenseNet	0.700	0.857
SqueezeNet	0.689	0.850
MobileNet	0.672	0.833
ShuffleNet	0.657	0.839
MobileViT	0.664	0.841
SqueezeVit (proposed model)	**0.703**	**0.851**

Table 8 records a cross-dataset comparison of the different models using the NIH and CheXpert datasets in terms of their AUROC. The findings point to the generalization ability of the models in the case when they are introduced to totally unseen data. It is observed that classical architectures such as DenseNet and SqueezeNet obtain 0.700 AUROC when trained on NIH and 0.690 when trained on CheXpert. Nonetheless, the performance reduction is observable in MobileNet and ShuffleNet, which implies that the lightweight models are less robust. Notably, the proposed SqueezeVit demonstrates consistent performance, achieving 0.703 AUROC on NIH and 0.694 on CheXpert, and maintaining competitive results when trained on CheXpert and tested on NIH (0.771 AUROC) and CheXpert (0.850 AUROC). Results obtained emphasize the fact that SqueezeVit generalizes better across datasets as compared to traditional lightweight baselines, filling the gap between the training and validation domains more precisely.

**Table 8:** Cross-Dataset AUROC Comparison for Various Models Trained and Tested on NIH and CheXpert.

Model	Tested on NIH		Tested on Chexpert	
	NIH	CheXpert	NIH	CheXpert
ResNet	0.688	0.681	0.762	0.849
DenseNet	0.700	0.690	0.776	0.857
SqueezeNet	0.689	0.689	0.777	0.850
MobileNet	0.672	0.634	0.718	0.835
ShuffleNet	0.657	0.644	0.681	0.831
MobileViT	0.664	0.652	0.750	0.840
SqueezeVit (proposed model)	0.703	0.694	0.771	0.850

**Fig. 8:**
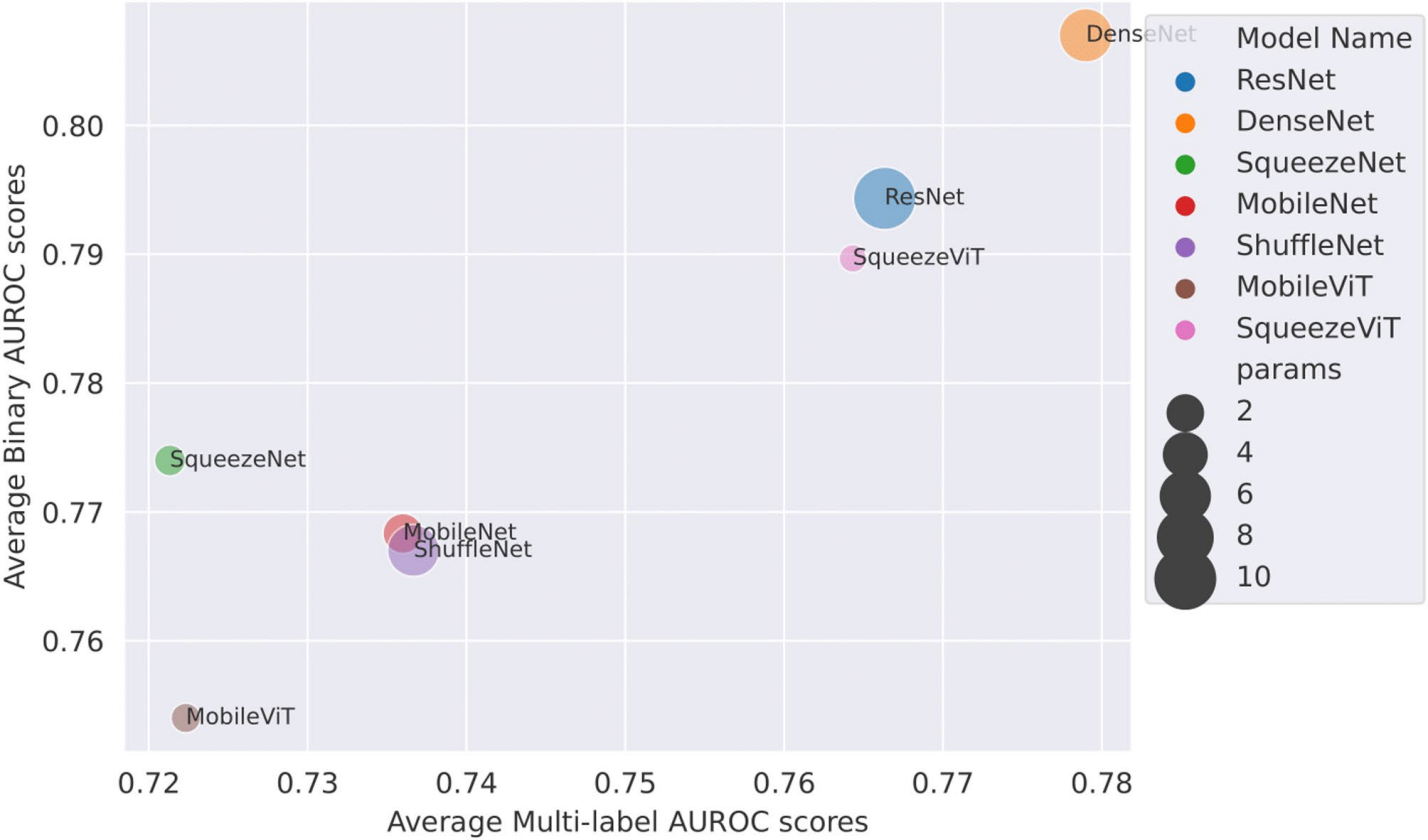
Comparison of models based on average binary and multi-label AUROC Scores with parameter size indication.

Binary and Multi-label AUROC scores with parameter size indication, the trade-off between prediction performance and the complexity of the models for different models are summarised by the plot depicted in Fig. 8. Our model reports competitive or SOTA eclipsing AUROC scores on binary/multi-label seizure prediction tasks, while maintaining the substantially smaller parameter size. This trade-off suggests that the proposed model is not only very accurate but also computationally efficient, which is essential for real-time and limited-resource clinical applications. The findings imply that it can effectively learn robust representations not only superior to the large architecture but also comparable to delicate training schemes.

## Ablation Studies

We conduct several ablation studies to study the importance of different components in our approach. In the experiments, we focused on multilabel classification. We report the mean AUROC of different ablation studies by both datasets to compare with the local and global explanations of our method.

### On the Importance of the Translution Block

In this ablation, the Translution Block is excluded from the original model architecture. The results show that there is a drop in performance as the score of the AUROC of NIH has decreased from 0.733 to 0.722. The findings are similar in all datasets. This indicates how the Translution Block can enhance the multilabel classification capabilities of SqueezViT. These ablation studies provide insightful hints on the importance of every element in the SqueezeViT model, as presented in Table 9. Although the model still has a competitive performance when some blocks are removed, it is clear that each of them influences the overall performance of the model.

It should be mentioned that Translution Block and Fire Block (*N* = 4) are stages that play a critical role in the proposed architecture, as stage 1 and stage 3. The lack of either of these blocks has a significant effect on the computational power of the model. However, it is possible to note that the absence of one of the stages is compensated by the other block, which proves the resilience of the model to ablation.

**Table 9:** Impact of Translution and Fire Block removal on Average AUROC for NIH and CheXpert Datasets.

Ablation	NIH	CheXpert
w/o TranslutionBlock	0.722	0.732
w/o Fire Block (*N* = 1)	0.732	0.747
w/o Fire Block (*N* = 4)	0.728	0.743
w/o Fire Block (*N* = 8)	0.725	0.738
w/o Fire Block (*N* = 16)	0.723	0.735
Proposed model	0.733	0.751

## Conclusions

In summary, SqueezeViT shows promising prospects in the CXR classification task with efficient computation and competitive accuracy to the SOTA methods. The proposed architecture employs the Fire Block for local representation learning and the Translution Block for efficient global representation learning. Although the network is very lightweight, the AUROC scores of SqueezeViT are on par with those of the larger models, such as ResNet and DenseNet, which means that it effectively extracts informative instances but with smaller complexity. Because only a few parameters and little computation are required, the model is extremely suitable to be utilized in memory-limited settings without sacrificing classification performance. This shows the SqueezeViT’s potential of serving as a light-weight alternative to traditional deep CNNs in the image-based task on the large-scale. The limitations of the datasets include label noise from automated annotations, class imbalance across disease categories, and domain bias from institution-specific data. Further, it is intended to explore the possibility of multitasking learning by considering suitable modifications of the proposed framework to address simultaneous localization and classification of abnormalities in CXRs in future work. Furthermore, we will investigate the extension of the proposed model to diagnose other radiological images, such as CT and MRI.

## Data Availability

The datasets used in this work are available at: [https://www.kaggle.com/datasets/nih-chest-xrays/data](https:/www.kaggle.com/datasets/nih-chest-xrays/data)[https://aimi.stanford.edu/datasets/chexpert-chest-x-rays](https:/aimi.stanford.edu/datasets/chexpert-chest-x-rays)The model of the proposed SqueezeViT is available on the link: [https://github.com/vijay13787/SqueezeVIT.git](https:/github.com/vijay13787/SqueezeVIT.git).
